# Depression core network-based individualized targeting for transcranial magnetic stimulation

**DOI:** 10.1016/j.brs.2023.03.005

**Published:** 2023-03-15

**Authors:** Tuukka T. Raij, Emma Komulainen, Dogu Baran Aydogan, Siina Pamilo, Erkki Isometsä, Tommi Raij

**Affiliations:** a Department of Psychiatry, University of Helsinki and Helsinki University Hospital, P.O. Box 590, FI-00029, HUS, Helsinki, Finland; b Department of Neuroscience and Biomedical Engineering, and Advanced Magnetic Imaging Center, Aalto NeuroImaging, Aalto University School of Science, P.O Box 13000, FI-00076, AALTO, Espoo, Finland; c A.I. Virtanen Institute for Molecular Sciences, University of Eastern Finland, P.O. Box 1627, FI-70211, Kuopio, Finland; d Athinoula A. Martinos Center for Biomedical Imaging, Department of Radiology, Massachusetts General Hospital, Charlestown, MA, USA; e Department of Radiology, Harvard Medical School, Boston, MA, USA

**Keywords:** Transcranial magnetic stimulation, Targeting, Functional magnetic resonance imaging, Major depressive disorder, Emotion regulation

## Abstract

**Background:**

Transcranial magnetic stimulation (TMS) of the dorsolateral prefrontal cortex (DLPFC) is an established treatment for major depressive disorder (MDD). Recent attempts to improve TMS efficacy by individually targeting DLPFC subregions that are functionally connected to the subgenual anterior cingulate cortex (sgACC) appear promising. However, sgACC covers only a small subset of core MDD-related areas. Further, fMRI connectivity of sgACC is poorly repeatable within subjects.

**Methods:**

Based on an fMRI database analysis, we first constructed a novel core network model (CNM), capturing voxelwise emotion regulation- and MDD-related DLPFC connectivity. Then, in a sample of 15 healthy subjects and 29 MDD patients, we assessed (i) within-subject repeatability of the DLPFC connectivity patterns computed from time segments of varying lengths of individual-level fMRI data and (ii) association of MDD severity with the individual DLPFC connectivity strengths. We extracted group-level connectivity strengths in CNM from individual DLPFC coordinates stimulated with neuronavigated TMS in a separate sample of 25 MDD patients. These connectivity strengths were then correlated with individual TMS efficacy.

**Results:**

Compared with sgACC connectivity, CNM increased intraindividual repeatability 5-fold. DLPFC connectivity strength from CNM was associated with MDD severity and TMS efficacy. While the locations of CNM-based individual TMS targets remained constant within individuals, they varied considerably between individuals.

**Conclusions:**

CNM increased repeatability of functional targeting to a clinically feasible level. The observed association of MDD severity and TMS efficacy with DLPFC connectivity supports the validity of the CNM. The interindividual differences in target locations motivate future individualized clinical trials leveraging the CNM.

## 1. Introduction

Transcranial magnetic stimulation (TMS) is a promising treatment in several brain disorders, including major depressive disorder (MDD), which is among the leading causes of disability worldwide [1]. In MDD patients failing to respond to first-line treatments, TMS of the dorsolateral prefrontal cortex (DLPFC) is an established and well-tolerated treatment [2]. Meta-analyses suggest, however, that with current treatment protocols the majority of treatment-resistant patients do not respond to TMS [3,4].

Suboptimal targeting of core neuronal networks underlying MDD may be a major reason for limited efficacy [5]. For targeting, the TMS coil is typically positioned over the DLPFC area based on external scalp measures [6]. As functional DLPFC anatomy may vary between individuals with respect to scalp measures [5,7], several studies have attempted to develop individualized targeting methods that are based on resting-state functional MRI (fMRI) connectivity [5,7-15]. The overall conclusion of these studies has been that the clinical outcomes correlate with functional connectivity strength between the individual TMS target and a seed region [7,9,13-15]. Typically, this seed (*i.e.,* a region to compare fMRI time course with those from other locations in the brain) has been the subgenual anterior cingulate cortex (sgACC), as the sgACC has been associated with outcomes of several MDD treatments [13]. The fMRI signal-to-noise ratio is, however, poor in the sgACC regions due to susceptibility artifacts, contributing to poor reproducibility of sgACC connectivity-based targets at the individual level [16].

Recently, sgACC connectivity-based targeting of TMS in MDD has yielded promising clinical outcomes [11,12]. In these studies, targeting was, however, combined with a very intense TMS protocol and not compared with scalp-based targeting. To support such comparisons, feasibility of techniques for functional individualized targeting needs to be ascertained, including increasing validity for functioning of the core networks underlying MDD (referred to as network validity) and enhancing repeatability of individualized target localization.

There are many alternatives to map treatment targets. While data-driven approaches help to avoid some potential errors, results have been poorly reproducible with currently available sample sizes [17,18]. In accordance, results of meta-analyses on brain correlates of MDD have varied considerably [19]. One option to restrict problems related to multiple comparisons is to focus analyses based on clinical features. For example, while MDD is associated with many symptoms, its two key features are low mood and inability to feel pleasure [20]; other symptoms can vary, but without one of these features MDD cannot be diagnosed. Mood is a prevalent emotional state that is coupled to more fleeting emotions, linking the two fundamental MDD symptoms with emotion regulation, and accumulating evidence points to dysfunctional emotion regulation strategies in MDD [21]. At the neuronal level, the cingulo-opercular network, which is structurally and functionally altered in MDD and several other common psychiatric disorders [22], closely resembles the network most strongly associated with behavioral and emotion regulation [23]. Accordingly, developers of TMS treatment for MDD have suggested focusing the stimulation on brain systems supporting emotion regulation [24]. These findings motivated the construction of a core network model (CNM) in the present study.

The sgACC is involved in emotion processing [25], but according to current views, any single brain region or connection could cover only a small subset of the networks involved in emotion regulation in MDD [23,25-28]. Fox and coworkers suggested expanding the seed region to cover the entire brain, weighting each seed voxel in computation of connectivity with the voxel's connectivity with i) the sgACC or ii) effective TMS sites [29]. Results from such a seed map method have been reproducible [8] and the resulting targets have been shown to be associated with the clinical outcome of TMS treatment [7]. It remains, however, unknown whether the sgACC connectivity-based approach is optimal, and the field may benefit from consideration of other network models as well.

Meanwhile, knowledge about the brain correlates of MDD and emotion regulation has increased, enabling construction of models with potentially stronger network validity. MDD-related networks overlap widely with emotion regulation networks, including i) emotion-processing regions, such as the amygdala and the sgACC, and ii) regions that regulate these emotion-processing regions, such as the DLPFC and the cingulo-opercular network [23,25-28,33,34]. Existing human brain imaging databases enable voxelwise localization of relevant subregions and their connectivity [35], which can be used to improve target network validity. Further, delivering stimuli that engage emotion regulation instead of resting-state during fMRI might increase validity for emotion regulation. For example, emotional stories are an engaging naturalistic stimulus [36-38], which may increase repeatablility of fMRI [39].

Another major challenge of fMRI-based individualized targeting is the noisy signal, which leads to poor within-subject repeatability of the targets [16]. Attempts to enhance repeatability of sgACC connectivity with spatial clustering and smoothing reduce accuracy of the target maps [8,16,29]. While large network models may enhance repeatability [29], their validity for the targeted disorder and TMS efficacy remains poorly known. Accurate and repeatable targeting methods with enhanced network model validity would be necessary not only to define target locations, but also to estimate whether target locations vary between individuals to an extent that TMS targeting needs to be individualized [30-32].

Here, we aimed to increase network validity by constructing a novel CNM to capture MDD- and emotion regulation-related DLPDC connectivity. We hypothesized that this approach increases within-subject repeatability of fMRI connectivity-based DLPFC targets to a clinically feasible level, and that the resulting DLPFC connectivity reflects MDD severity and TMS outcome. Moreover, we hypothesized that presenting emotionally engaging audio stories during fMRI, instead of recording resting-state fMRI data without stimuli, would enhance the model performance. Finally, as repeatability depends on scan time, we assessed the effect of scan length on repeatability, with the overall aim of developing a feasible individualized functional targeting method for clinical use.

## 2. Materials and methods

### 2.1. Participants

Data from three separate studies were used for evaluating the network model. The first of these (“MDDAD”) included 29 MDD patients randomized to receive 10 mg of escitalopram (n = 15) or placebo (n = 14) daily for one week prior to imaging to evaluate early medication-related brain functions [36]. The second sample (“HC”) included 15 healthy controls without medication [37]. The third dataset included 25 TMS-treated MDD patients from a study of Weigand et al. (“MDDTMS”) [15] (See Supplementary Material).

### 2.2. Seed definition for the core network model

**Fig.1.**
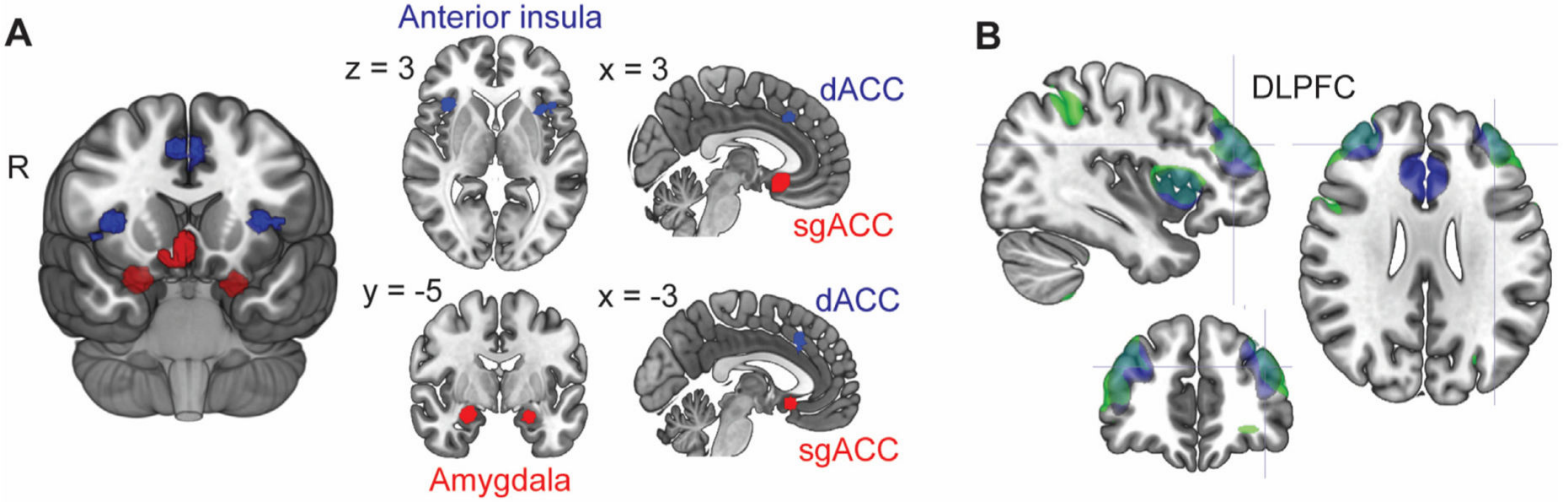
Seed regions of CNM and their DLPFC connectivity. Panel **A** shows seed region connected positively (blue) and negatively (red) with the DLPFC. Green in panel **B** presents sgACC connectivity and blue CNM connectivity (overlap in turquoise). Crosshair points to a previously suggested connectivity-based group-level TMS target in the left DLPFC (x, y, z = −38, 44, 26) [13]. dACC, dorsal anterior cingulate cortex; sgACC, subgenual enterior cingulate cortex; DLPFC, dorsolateral prefrontal cortex, CNM, core network model, R, right side.

To construct the CNM, we used Neurosynth, which is a large fMRI database that allows associating keywords with brain activation patterns [35]. This network model was constructed a priori, before testing it with the individual level data. To derive seed regions associated with MDD and emotion regulation, we constructed two probabilistic maps of voxels, the first one associated with the keywords “major depression” and the second associated with “emotion regulation”. Next, we multiplied these two maps, resulting in regions associated both with MDD and emotion regulation. As brain imaging studies on emotion regulation typically evoke emotions, the emotion regulation-related regions include both the regulatory and the regulated regions. As a regulatory region, the DLPFC has positive connectivity with other regulatory regions, such as the cingulo-opercular network, and negative connectivity with the regulated regions (especially in standardized connectivity maps), such as the amygdala and the sgACC. If connectivity with the DLPFC was summed from the regulatory and regulated regions, connectivities of these regions might cancel each other out. We thus differentiated the negatively and positively connected regions based on group-level functional connectivity with DLPFC in Neurosynth resting-state fMRI connectivity data of 1000 individuals. The DLPFC was defined here as the largest clusters of voxels in Neurosynth associated with the keyword “DLPFC”, multiplied with the Harvard-Oxford cortical (binary) mask. Both the left and the right DLPFC were included because both are used as TMS targets. We then selected as seeds the largest clusters of voxels associated with MDD and emotion regulation that showed positive or negative DLPFC connectivity (maximum correlation >0.1 or < −0.1 within the DLPFC mask) and agreed with the literature on emotion regulation and MDD [23,26-28]. The resulting clusters were located bilaterally within the amygdala and the cingulo-opercular network. In addition, even though sgACC, as defined by Fox et al. [13], did not meet the inclusion criteria for network nodes (existing association with “depression” but absent association with “emotion regulation”), this area was added post hoc to build a network that is more comprehensive for TMS targeting in MDD. Specifically, the rationale for including sgACC was several previous studies linking it to emotion regulation [25] as well as its central role in MDD [26,27,34] and TMS targeting in MDD [5,13]. Due to potential inter-hemispheric differences in brain functioning, seeds crossing the midsagittal plane were separated to left and right hemisphere parts, resulting in a total of eight seeds in non-DLPFC regions (Fig. 1 and Table 1). Supplementary Figure 1A shows differences together with considerable overlap of MDD-related and emotion-regulation related regions in the Neurosynth data.

### 2.3. TMS treatment

We used the published clinical data set of 25 patients who completed TMS treatment for MDD (MDDTMS) [15] to compare CNM connectivity with clinical outcomes. Eighteen patients received standard 10-Hz stimulation and 7 patients received 20-Hz stimulation. Eleven patients were treated with the Magstim^®^ and 14 with the Neuronetics^®^ TMS system, and the number of treatment sessions ranged from 20 to 32. MRIs were acquired before treatment and coil position was registered with the Brainsight^®^ Neuronavigation system (see original publication for further details).

### 2.4. Imaging and stimulus

We had imaging data from the MDDAD and HC samples (but only stimulation coordinates for the MDDTMS sample). The functional blood-oxygenation-level-dependent (BOLD) and T1-weighted structural MRIs were acquired at 3T (Supplementary Material) in our earlier studies [36,37]. In MDDAD and HC each subject's fMRI data with “stories” consisted of time series of 1000 full volume acquisitions that had been recorded while presenting a total of 30 audio stories. Neutral stories and stories with negative and positive emotional content, ten of each three categories, and 45 s of duration each, were presented with the Presentation^®^ software (Neurobehavioral Systems Inc., Albany, CA, USA). Valence of the stories was confirmed with subjective ratings in an earlier study [38]. The resting-state fMRI data were available from the MDDAD patients only (not from the HC dataset) and consisted of 250 full-volume acquisitions without stimuli. The data were acquired during the same MRI session before obtaining the data with “stories”. For further details, see Supplementary Material and references [37,38]. The MRI data from MDDAD and HC were also used to derive estimates of connectivity strengths in coordinates actually stimulated in the separate MDDTMS sample [15].

### 2.5. Preprocessing of individual data

We preprocessed the imaging data using the DPABI pipeline [40] and SPM12 (https://www.fil.ion.ucl.ac.uk/spm/software/spm12/). We used movement correction and spatial normalization of fMRIs into the Montreal Neurologic Institute template based on structural T1 images with a fast diffeomorphic image registration algorithm (DARTEL) [41]. We did not use spatial smoothing, as it increases repeatability at the cost of spatial accuracy. We regressed out movement parameters and their exponentials, CSF and white matter signals, bandpass-filtered signal time series at 0.01–0.1 Hz, and removed 1 previous and 2 later volumes at time points with framewise displacement (FD_Power) > 0.5 mm to reduce movement artifacts.

### 2.6. CNM to define targets at the individual level

We computed connectivity of the seed regions defined in 2.2 (see also Fig. 1) as Pearson's correlation between the seed fMRI time series and those in other voxels in the whole brain, but focused on the connectivity of the seed regions with the DLPFC due to its established role in TMS treatment of MDD. In the model, the DLPFC and the cingulo-opercular regions relate to downregulation of the negatively connected regions (sgACC and amygdala) that are involved in eliciting and experiencing emotional responses [25-27]. In such a network, a regulatory region may activate together with other regulatory regions (reflected in positive connectivity between regulatory regions) to down regulate activity of regulated regions (reflected in negative connectivity between the regulatory regions and the regulated regions). In this network, the connectivities of regulatory and regulated regions with a target region are thus opposite (i.e., have different sign). Combining connectivities with different signs would cancel each other out and result in biased targeting. Thus, our model combines sign-reversed negative connectivity of the sgACC and amygdalae and positive connectivity of the cingulo-opercular seeds. This was operationalized by multiplying the individual-level sgACC and the amygdala connectivity maps (images of voxelwise connectivity) by −1 (note that absolute values cannot be used here as this would mix positive and negative connectivity). Then the eight connectivity maps were averaged to one target map for each individual, reflecting voxelwise strength of emotion regulation-related DLPFC connectivity in the MDD-related networks. These maps were computed separately for resting state and story stimulus data. Supplementary Figure 1B shows both differences and overlap in connectivity of the MDD-related and emotion-regulation related regions.

### 2.7. Assessing within-subject repeatability of target maps

Individual target maps, within-subject spatial correlations between the target maps, and connectivity strengths were computed from the MDDAD and HC datasets in Python using standard libraries (e.g., pandas [42], numpy [43], scipy [44]) and Nilearn [45] that relies upon scikit-learn [46]. We split the fMRI time series to segments of equal length and compared target maps derived from the separate segments within individuals. To assess the dependency of repeatability on scan length, we used data segments of different lengths. Specifically, the 1000 vol collected during stories were split into eight segments of 125 images, 4 segments of 250 images, and 2 segments of 500 images. The 250 vol collected during rest were split to two segments of 125 images. Next, we computed the target maps from each segment separately for each participant. Then, we computed intraindividual spatial correlation (*r*_spat_) across all the individual maps computed from the same length of scan (e.g., an average of spatial correlation between all pairs of the maps computed from four fMRI data segments of 250 vol each). We focused on the DLPFC target regions used in TMS treatment in MDD, computing *r*_spat_ within the bilateral DLPFC mask. We used Fisher Z-transformation before two-tailed statistical tests on correlation values.

As the relationship between scan time and repeatability of connectivity follows an exponential function [47], we fitted the function *r*_spat_ = *r*_spatmax_–e^(−t/a)^ to the data to estimate *r*_spat_ across the different scan lengths [48]. Here, e and *a* are constants, *r*_spatmax_ is the maximum achievable *r*_spat_ with the present data, and *t* is the scan time.

### 2.8. Comparing repeatability between models and stimulus type

For fMRI data recorded with stories in the MDDAD and HC datasets, we computed *r*_spat_ for 7 min (250 vol) and 14 min (500 vol) using sgACC only *vs*. the entire CNM. We then compared *r*_spat_ between the two models with equal scan durations (250 *vs*. 250 vol and 500 *vs*. 500 vol). For resting-state fMRI data, available in MDDAD, we computed *r*_spat_ using the CNM for a data segment of 3.5 min (125 images as longer repeated segments were not available) and then compared it with *r*_spat_ for equally long data segments recorded with stories.

### 2.9. Comparing target locations across individuals

Using the MDDAD and HC data, we selected 100 DLPFC voxels with the largest connectivity values in the CNM for each subject for each hemisphere (referred to as targets) computed individually (see section 2.6) from 1000 functional volumes during stories. The resulting maps were binarized, summed together, divided by the number of subjects, and multiplied by 100, resulting in a percentage map of common target voxels between individuals. We also computed the Euclidean distance between the most strongly connected voxels across individuals in the left DLPFC.

### 2.10. Comparing target connectivity strengths between stimuli and groups

To test the validity of the model in independent samples, the connectivity strengths of individual targets (computed as average connectivity across the 100 target voxels and 1000 fMRI volumes, referred to as target connectivity strengths) were compared across individuals between MDDAD and HC and correlated with severity of MDD in the MDDAD sample. Here, target connectivity values were pooled across the left and right hemispheres because they were strongly intercorrelated (r = 0.91, P = 3*10^−17^). MDD severity outcomes comprised of Montgomery-Åsberg Rating Scale (MADRS) [49] and Beck's Depression Inventory (BDI) [50] total scores, averaged across 0 and 1 weeks before fMRI.

### 2.11. Comparing model connectivity strengths with TMS clinical outcomes

We used published individual-level data of stimulation coordinates and BDI % change before vs. after treatment in the MDDTMS sample [15]. These coordinates were defined as the nearest cortical location from the coil center during treatment. CNM connectivity values for the coordinates were derived for each individual in the MDDTMS group from the average across the individual CNM connectivity maps of the MDDAD patients. We then correlated the group level CNM connectivity strengths from individual stimulation locations with the TMS-related BDI % change across individual MDDTMS patients following Fox and coworkers [13]. These correlations were adjusted for device, stimulation protocol, and number of treatment sessions. For comparison, we correlated the TMS outcome with the sgACC, emotion-regulation, and MDD-related connectivity separately.

## 3. Results

### 3.1. Sample characteristics

Supplementary Table 2 shows the sample characteristics. MDDAD and HC samples [36,37] did not differ significantly in sex, but differed in age. Thus, age was adjusted for when comparing these groups. In the MDDAD group, BDI score was 25 ± 8. In MDDTMS data [15], BDI score was 39 ± 9 before and 21 ± 13 after treatment.

### 3.2. Comparison of within-subject repeatability of the target maps between CNM and sgACC connectivity

**Fig. 2.**
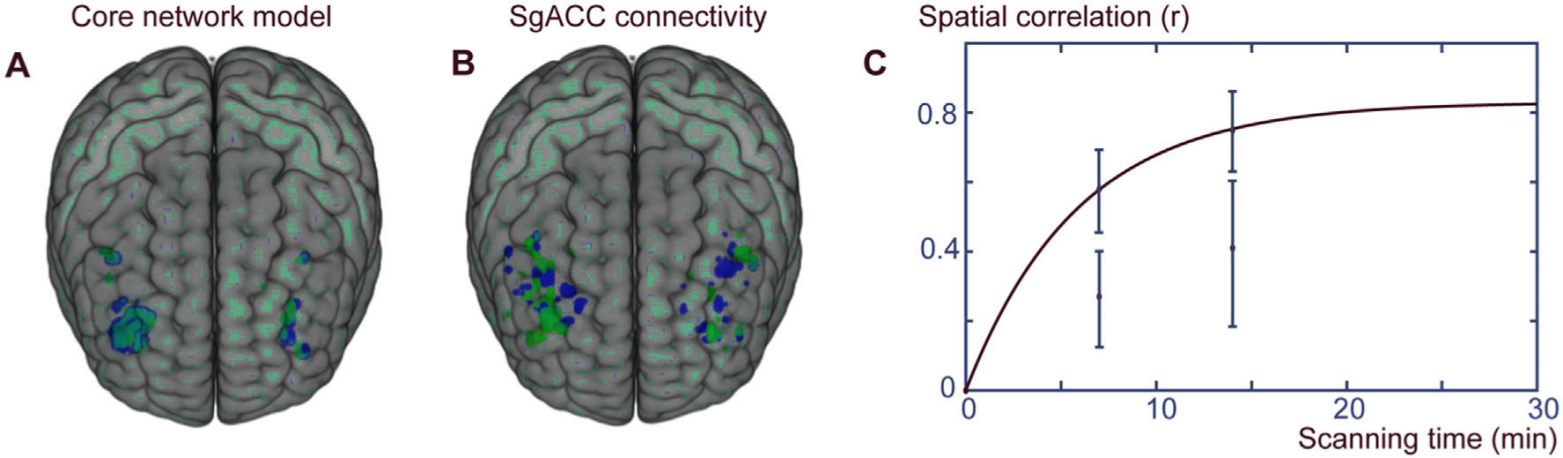
Intraindividual repeatability of target maps for CNM and sgACC connectivity. Panels **A** and **B** show intraindividual repeatability of target maps for a representative individual for CNM and sgACC connectivity, respectively. Green shows target maps from one 14-min fMRI data segment and blue from another 14-min fMRI data segment in the same individual during stories. Panel **C** presents dependency of repeatability on scan time. Error bars present ±1 SD for CNM (upper bars) and for sgACC connectivity (lower bars) at 7 and 14 min of fMRI data acquisition during stories. An exponential function was fitted to the CNM data to evaluate repeatability at different scan lengths. CNM, core network model; sgACC, subgenual anterior cingulate cortex.

These analyses focused on the DLPFC. We first compared repeatability using within-subject spatial correlation (*r*_spat_) of the target maps computed from juxtaposed 7-min fMRI-data segments during stories. As the difference in *r*_spat_ between HC vs MDDAD was non-significant (P > 0.22), the analysis of repeatability for stories (available for both groups) was conducted in pooled groups in addition to MDDAD group. Compared with sgACC connectivity alone (*r*_spat_ = 0.27 ± 0.14 and 0.26 ± 0.14, for pooled and MDD groups, respectively), the CNM resulted in a clearly higher *r*_spat_ (0.58 ± 0.14 and 0.59 ± 0.12, P = 6.1 × 10^−14^, and 8.2 × 10^−12^ for the pooled and MDDAD group, respectively, paired samples *t*-test). Variance of a target map constructed from one data segment explained by those constructed from other data segments (*i.e.,* $r_{\text{spat}}$) was 4.5- and 5.1-fold higher (for the pooled and MDDAD group, respectively) when computed between consequent story segments and 5.5-fold higher when computed between the story and the resting-state segments of equal length (MDDAD group). Examples of individual targets with the two models are presented in Fig. 2A and B, and Fig. 2C shows *r*_spat_ dependence on scan time.

### 3.3. Within-subject repeatability of the target maps depending on scan time and stimulus type

With stories as stimuli, the spatial correlation of the CNM target maps increased from *r*_spat_ = 0.58 ± 0.14 and 0.59 ± 0.12 at 7 min to *r*_spat_ = 0.75 ± 0.12 and 0.76 ± 0.08 for the pooled and MDDAD group, respectively, at 14 min segments. For resting-state fMRI in MDDAD sample, the spatial correlation of target maps from 7 min story segments was 0.49 ± 0.11. For story stimulus, the best fit for the dependency of *r*_spat_ on scan time was achieved with an exponential function with maximum achievable group average of *r*_spat_ = 0.83 during stories.

The stories tended to result in higher *r*_spat_ than rest (*r*_spat_ = 0.36 ± 0.11 *vs*. 0.31 ± 0.12 for 3.5 min that were the longest available repeated segments for resting-state data, P = 0.06). Mean framewise displacement was similar during the stories (0.09 ± 0.04 mm) and rest (0.08 ± 0.03 mm; P = 0.52).

### 3.4. Interindividual differences between targets

**Fig. 3.**
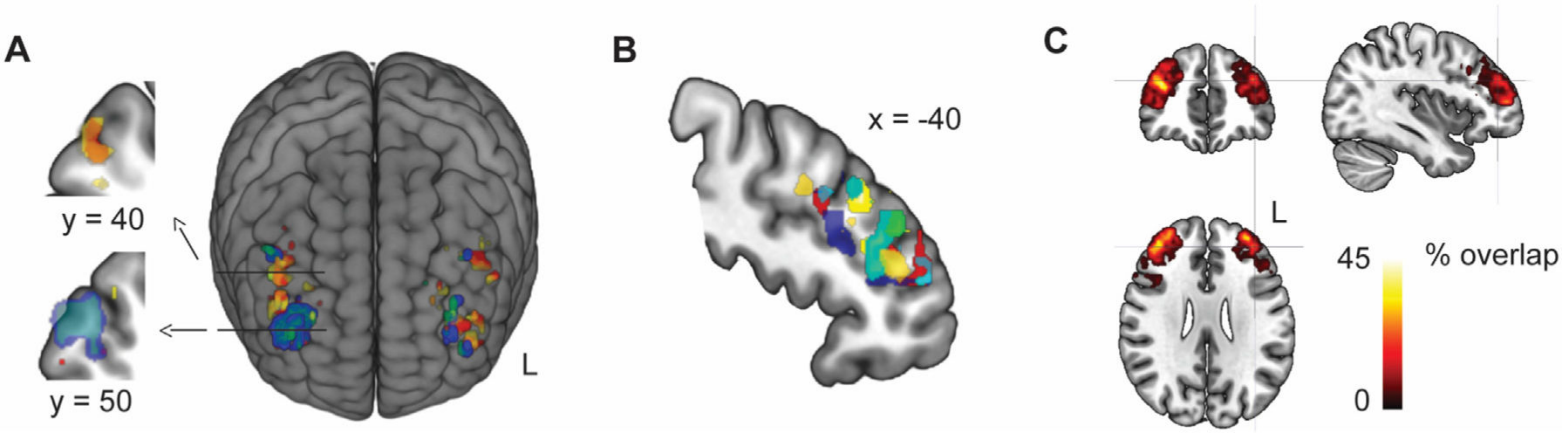
Intraindividual vs interindividual variance of the CNM target locations. In panel **A**, red/yellow show target maps computed from two 14-min segments of fMRI data in one individual (overlap in orange) and blue/green in another individual (overlap in turquoise). Panel **B** shows CNM targets in eight randomly selected subjects on a sagittal slice. Different colors represent different subjects computed from 28 min of individual-level fMRI data. Panel **C** shows percentual intersubject overlap of targets across all 29 MDDAD patients during stories. Individual targets consist of 100 voxels for each hemisphere. The crosshair points to the connectivity-based group-level target suggested by Fox and coworkers [13].

Group average of the target maps from the CNM during stories in the MDDAD sample matched well with an earlier estimate of the most efficient stimulation site in the left hemisphere (x, y, z = −38, 44, 26) [13]. However, this group-level result did not represent the individual-level maxima well because this voxel was among the 100 strongest individual DLPFC connections in the CNM in only 28% of the patients. The most strongly connected voxels tended to cluster within each individual, and the locations and shapes of these clusters differed clearly between individuals (Fig. 3). The strongest overlap was located at x, y, z = 36, 45, 24, which was among the top 100 individual target voxels in 41% of the patients (Fig. 3). The between-subject Euclidean distance between the most strongly connected voxels was 18 ± 4 mm (mean ± SD) in the left hemisphere.

### 3.5. Comparison of CNM target connectivity strengths between stimuli and groups

**Fig. 4.**
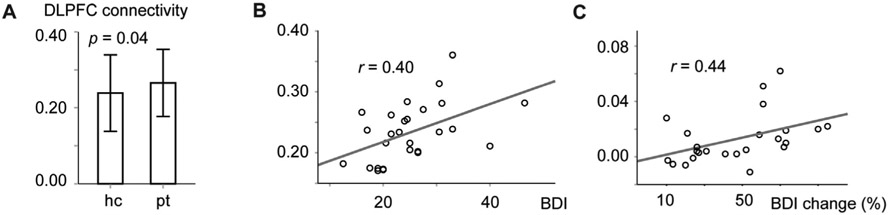
Relationship of the core network model target connectivity strengths with depression and effect of TMS. Panel **A** shows difference between patients (pt) and healthy control subjects (hc) in the target connectivity strengths during stories. Panels **B** and **C** present correlation of the connectivity strengths during rest with depression severity and TMS response, respectively. BDI, Beck's Depression Inventory; BDI change %, percentual decrease in BDI during TMS treatment. Error bars represent ±1 SD.

Target connectivity strengths from the CNM in the MDDAD group were stronger while listening to the stories than during rest (*r* = 0.27 ± 0.04 *vs*. 0.23 ± 0.05, P = 0.002; paired samples *t*-test; note that connectivity strengths do not depend on scan time as repeatability does). Fig. 4A shows that the patients (MDDAD) had stronger target connectivity than the healthy controls (HC) (*r* = 0.27 ± 0.05 *vs*. 0.24 ± 0.05) during stories (P = 0.04). There was no difference between the medicated and unmedicated patients in connectivity strengths during stories or rest (P > 0.91).

### 3.6. Correlation of target connectivity strengths with MDD severity

Fig. 4B shows that during rest the target connectivity strength correlated with depression severity (*r* = 0.40, P = 0.03) as assessed with BDI. No such correlation was observed during stories or with MADRS (*P* > 0.05). sgACC connectivity strength did not differ between patients and control subjects; it also did not correlate with symptom severity during stories or rest (p > 0.25).

### 3.7. Correlation of target connectivity strengths with clinical response to TMS

Fig. 4C shows that the MDDAD group's average connectivity strengths from CNM, extracted from the coordinates of the MDDTMS patients targeted during TMS treatment, correlated (*r* = 0.44, P = 0.03 during rest; *r* = 0.48, P = 0.02 during stories) with the treatment outcomes, as measured with BDI. These correlations survived adjusting for device, stimulation protocol, and number of treatment sessions (partial correlation P < 0.05). Correlations of TMS outcome with connectivity of emotion regulation and MDD-related regions, computed separately, were r = 0.32, P = 0.12; r = 0.37, P = 0.07 respectively, during stories. No correlation with TMS outcome was observed with sgACC connectivity alone (P > 0.3 during stories and rest).

## 4. Discussion

We constructed a novel network model with strong face validity for MDD-related core networks and showed the resulting targets to be repeatable within individuals and valid for MDD and the TMS treatment response in MDD in independent samples.

The finding that the cingulo-opercular regions and the DLPFC were negatively connected with the sgACC and amygdala is not new. The cingulo-opercular regions and the DLPFC are, however, large and functionally heterogeneous regions. The CNM seeds were localized based on a large database to the cingulo-opercular subregions most strongly associated with MDD and emotion regulation. Thus, connectivity of these subregions may help to advance localization of TMS-targets for MDD.

The CNM, based on association with the keywords “major depression” and “emotion regulation” as well as DLPFC connectivity in the Neurosynth database, agrees well with the previous literature. MDD relates to an imbalance between the cortical (regulatory) and the deep emotion-related (regulated) systems centered at the amygdala and the sgACC [26,27]. Furthermore, recent findings suggest that the most important cortical emotion regulation circuitries include the anterior insula and the dorsal anterior cingulate cortex [23]. These cingulo-opercular regions have direct structural connections with the DLPFC, amygdala, and sgACC, being well-positioned to regulate the deep emotion-related systems and to mediate regulatory signals from the DLPFC [13,51].

Emotion regulation encompasses explicit and implicit processes that identify the need to regulate emotions, select among available regulatory strategies, implement the selected strategy, and monitor the outcome [52]. The present model may benefit further studies aimed at testing the long-held hypothesis that TMS effects in MDD are mediated by modulation of the emotion regulation pathways [24]. For this purpose, changes in different components of emotion regulation can be assessed by multiple neuropsychological and brain-level measures [25,52] and linked in causal models to treatment-related changes in symptoms and brain measures.

Patients had stronger target connectivity in the CNM than control subjects, and the target connectivity strengths were positively correlated with the BDI scores. This agrees with the previous literature suggesting that during automatic emotion regulation situations (such as when presented with emotional stimuli without an explicit emotion regulation task) patients with MDD tend to engage DLPFC more strongly than healthy control subjects, which may reflect compensatory mechanisms [25].

Adding to earlier findings of sgACC connectivity [7,9,15,29] our results suggest that functionally defined TMS targets differ considerably between MDD patients. The observed 18-mm mean difference between targets across individuals was larger than the error of a few millimeters resulting from modern non-linear spatial normalization to a common brain template [53-55]. Meanwhile, intraindividual repeatability was high, especially considering that no spatial smoothing was used. While smoothing of fMRI data increases apparent repeatability [16], it reduces spatial accuracy. Importance of the spatial accuracy of the target maps depends on the strength and spatial distribution of the TMS-induced electric fields needed to trigger therapeutic mechanisms, while such measures remain inadequately known [56-58]. However, induction of individual finger movements in humans as well as single-cell recordings in non-human primates suggest that TMS-induced action potentials can be restricted to a few square millimeters of the cortex [59,60]. If the presently observed spatial accuracy with non-smoothed data was unnecessary for some applications, smoothing of the data would increase repeatability considerably beyond the present results [16] and allow even shorter scan times.

The present results also suggest that employing emotional story stimuli during fMRI, as opposed to resting-state paradigms, may increase connectivity of the target maps and reduce scan time demands. On the other hand, the relatively high spatial correlation between story and resting-state target maps implies that resting-state data may be sufficient, provided that the scan time is adequate. As auditory stimuli such as stories can be easily presented in the MRI-scanner, we recommend further studies to consider the potential advantages of using auditory narratives.

Limitations of this study include relatively small samples, limited amounts of resting-state data, and the lack of individual-level brain imaging data of MDDTMS patients. This may explain the mixed findings on correlation between the CNM target connectivity strengths and the measures of MDD severity, as well as the trend-level difference in repeatability between the rest and story stimulus. While repeatability of connectivity from 3.5 min fMRI segments is generally poor, this depends on the analysis. The fitted function in Fig. 4C suggests that repeatability of the CNM-based targets is relatively good already at 3.5 min fMRI data. Notably, we used these short data segments only to compare repeatability and connectivity strengths of the CNM-based targets between rest and story stimulus, while other analyses used longer data segments.

Better understanding of the subprocesses of emotion regulation would enhance development of these lines of research. Even if the fundamental symptoms of MDD relate to emotion regulation [20], it is possible that at least some patient groups may benefit from separate targeting for different symptoms or symptom clusters such as anxiety and somatic symptoms [61]. Although we focused on the DLPFC, future research should consider potential targets in other regions as well. An alternative way to map seeds or targets related to MDD and emotion regulation would be to directly assess regions of altered emotion regulation in MDD patients. We selected the present way as a large amount of imaging data is considered necessary for reliable localization, and the available amount of data on MDD and emotion regulation was considerable larger than that on emotion regulation in MDD patients. Even if brain correlates of psychiatric disorders may be better explained as functions of networks than of a single region, future clinical trials are needed to show the difference in treatment outcome between different single- and multi-seed models. Prospective randomized controlled trials are also needed to exclude the possibility that such targeting models would relate to treatment resistance rather than to clinically useful localization.

The main strength of the study is its innovative use of individualized neural network connectivity patterns to identify targets for treatment of MDD. To our knowledge, there are no previous studies presenting a network targeting model with strong face validity for the fundamental emotion regulation-related core symptoms of depression, supported by association between the target connectivity and both the MDD symptoms scores and TMS outcome. Furthermore, our findings add to the literature showing that such a model associates with good repeatability and spatial accuracy, which may be further enhanced using a naturalistic stimulus during imaging. In conclusion, these findings suggest that repeatable and accurate individual targets can be computed based on connectivity of the emotion regulation-related core neuronal networks of MDD. The target connectivity strengths differed between MDD patients and healthy control subjects and correlated with MDD severity and TMS outcome in MDD in separate samples, supporting model validity. The distance between repeatable individual targets was considerable. Such findings build the foundation for future clinical trials to test whether and to what extent individual functional targeting enhances outcomes.

## Supplementary Material

1

## Figures and Tables

**Table 1:** Seed regions of the core network model.

Seed Region	Association with “Major Depression”				Association with “Emotion Regulation”				Connectivity with DLPFC			
		
	x	y	z	*Z*-score	x	x	z	*Z*-score	x	y	z	*r*
Left anterior insula	−34	20	4	11.7	−36	14	2	6.8	36	45	30	0.39
Right anterior insula	36	20	−4	11.7	36	20	0	9	45	39	27	0.34
Left dorsal anterior cingulate cortex	−2	24	40	9	−2	22	40	10.2	−33	54	18	0.34
Right dorsal anterior cingulate cortex	2	20	36	14.9	2	20	36	11.5	30	48	30	0.44
Left amygdala	−22	−6	−16	22.6	−22	−6	−20	10.2	33	51	30	−0.18
Right amygdala	22	−2	−16	17.9	26	−2	−20	10.6	36	54	24	−0.14
Left subgenual cingulate cortex	−6	16	−10	NA	−6	16	−10	NA	42	48	27	−0.15
Right subgenual cingulate cortex	6	16	−10	NA	6	16	−10	NA	39	48	30	−0.16

*Z*-scores indicate the association strengths with keywords “major depression” and “emotion regulation”, and *r* values the connectivity strengths of each seed with DLPFC (strongest connection is shown independently of laterality) in Neurosynth database. Coordinates refer to the voxel with the strongest association with the keywords or the strongest (negative or positive) connectivity with a seed region.
