# Supplementary material for: Depression core network-based individualized targeting for transcranial magnetic stimulation
Source: Brain Stimul. Author manuscript; Available in PMC 2023 Aug 7. (PMC10404717; doi:10.1016/j.brs.2023.03.005)
Supplement: 1 [file NIHMS1898327-supplement-1.docx]

**Supplementary Material**

Raij et al.

**Depression core network-based individualized targeting for transcranial magnetic stimulation**

**Participants**

The first data set (MDDAD) comprised 37 treatment-seeking MDD patients aged 18–65 years recruited from the Finnish Student Health Service at Helsinki and Espoo, Finland (see Supplementary Table 1 for inclusion and exclusion criteria) [1]. Data from 8 subjects were excluded from analysis (excess head movement during imaging n = 2, MRI scan failure due to technical problems n = 2, anxiety n = 1, drop-out from the study n = 3). In the second data set of 15 healthy control subjects (HC) [2] the exclusion criteria included any current or previous psychiatric disorder, use of illicit drugs, consumption of alcohol >24 U/week for men and >16 U/week for women and current use of antidepressant or antipsychotic agent, mood stabilizer, systemic corticosteroid, beta blocker, or benzodiazepine. The third data set (MDDTMS) including TMS outcomes and target coordinates was collected in Boston from treatment-seeking, medication-resistant MDD patients aged 28–67 years [3]. Exclusion criteria included psychosis, bipolar disorder, obsessive compulsive disorder, eating disorder and prior treatment with electro-convulsive therapy (see details from [3]). Eleven of 36 patients dropped out during treatment. Baseline characteristics of the three datasets are described in Table S2.

**MR imaging and story stimulus**

Images of MDDAD and HC were acquired with a 3 T MAGNETOM Skyra and a 20-channel Siemens receiver coil (Siemens Healthcare, Erlangen, Germany) at the Advanced Magnetic Imaging Center, Aalto NeuroImaging, Aalto University School of Science, Espoo, Finland. For fMRI, a blood-oxygenation-level-dependent (BOLD) echo-planar imaging (EPI) sequence was used to acquire 33 slices (axial oblique orientation according to paranasal sinuses, TR 1700 ms, TE 24 ms, FOV 202 mm, flip angle 70°, GRAPPA acceleration factor 2) and voxel size of 3×3×4 mm. Structural T1-weighted MRI was acquired with a MPRAGE sequence (TR 2530 ms, TE 3.3 ms) at a 1-mm isotropic spatial resolution.

We presented the stories read by a neutral female voice, with an UNIDES ADU2a audio system (Unides Design, Helsinki, Finland) via plastic tubes through porous EAR-tip (Etymotic Research, ER3, IL, USA) earplugs. Audio volume was adjusted individually to be clearly audible over scanner noise. We asked the participants to concentrate on listening to the stories and to imagine the events vividly. Each story was preceded by a 5 second fixation cross and a 15 second written introduction to the next story.

**Supplementary Table 1.** Inclusion and exclusion criteria of the MDDAD dataset.

| **Inclusion criteria** | **Exclusion criteria** |
| --- | --- |
| MDD in SCID I -interview | Antidepressant use 4 months prior to study |
| MADRS score 15­–30 | Psychotic symptoms |
|  | Borderline, schizotypal or schizoid personality disorder |
|  | Primary anxiety disorder |
|  | Previous suicide attempt |
|  | Severe suicidal ideation |
|  | Severe unstable somatic illness |
|  | Depression due to somatic illness or substance use |
|  | Life-time alcohol or drug dependence |
|  | Alcohol or drug abuse during the last 12 months |
|  | Consumption of alcohol >24 U/week for men and >16 U/week for women |
|  | Current use of illicit drugs |
|  | Contraindication for MRI |
|  | Current use of an antipsychotic agent, mood stabilizer,  systemic corticosteroid, beta blocker, or benzodiazepine |

These criteria consider the MDDAD dataset [1, 2]. MDD, major depressive disorder; MADRS, Montgomery-Åsberg Rating Scale [4]; SCID I, Structured Clinical Interview for DSM IV Axis I disorders [5]; MRI, magnetic resonance imaging.

**Supplementary Table 2.** Baseline characteristics of the three datasets used in the study.

|  | MDDAD | HC | MDDTMS |
| --- | --- | --- | --- |
| AGE mean (SD) | 27.1 (5.4)* | 24.0 (5.0)* | 54.8 (9.9) |
| SEX male/all | 13/29 | 3/15 | 8/25 |
| AD medication | 15/29 | 0/15 | 25/25 |
| BDI mean (SD) | 25.2 (7.5) |  | 38.6 (9.3) |
| MADRS | 22.6 (4.2) |  |  |

AD medication, antidepressant medication; BDI, Beck Depression Inventoy; MADRS, Montgomery-Åsberg Rating Scale. *p<0.001 in Kruskall-Wallis test. No comparisons shown for MDDAD vs MDDTMS, as we did not compare these groups in the present study.

**A**


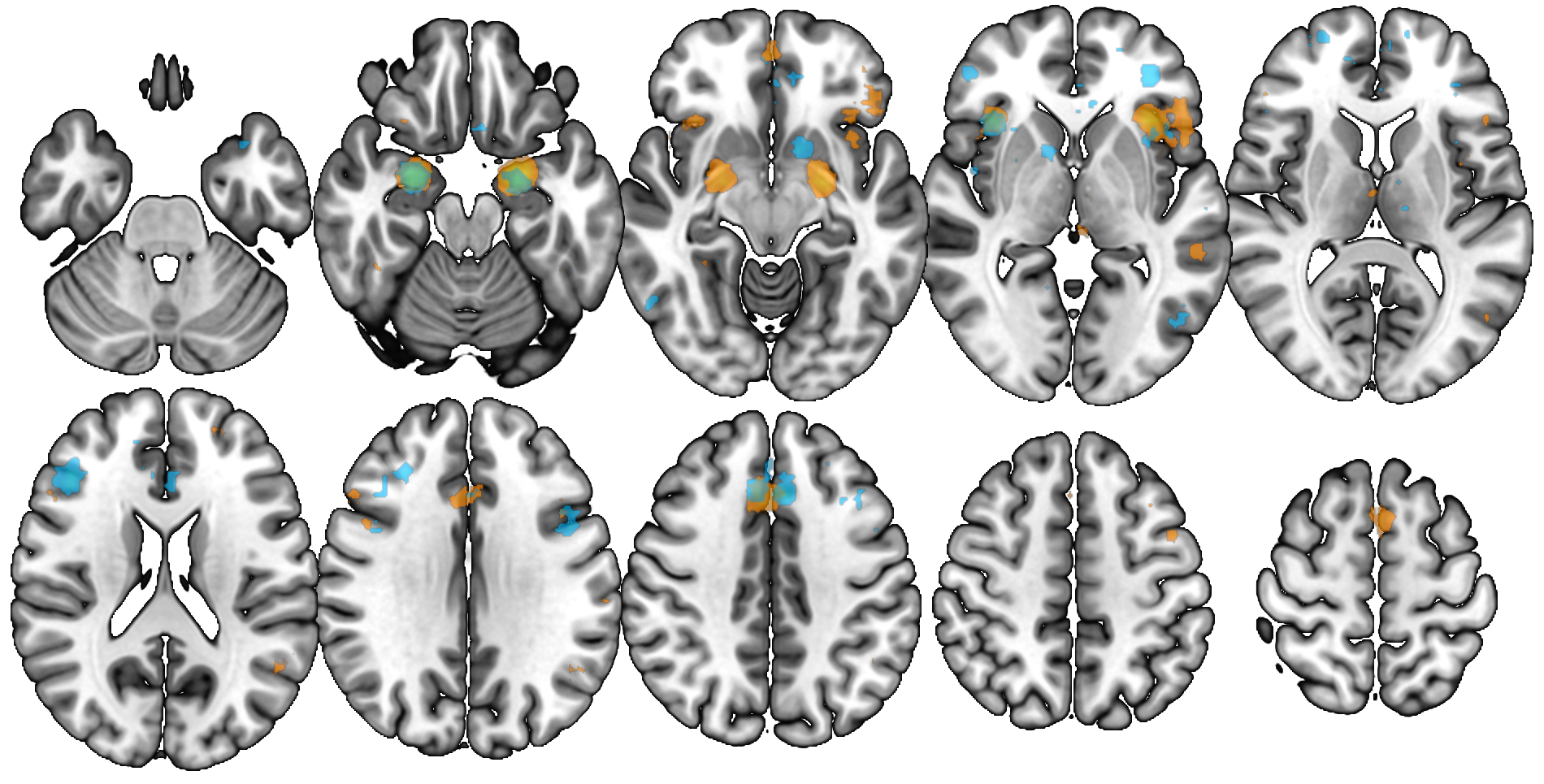


**B**


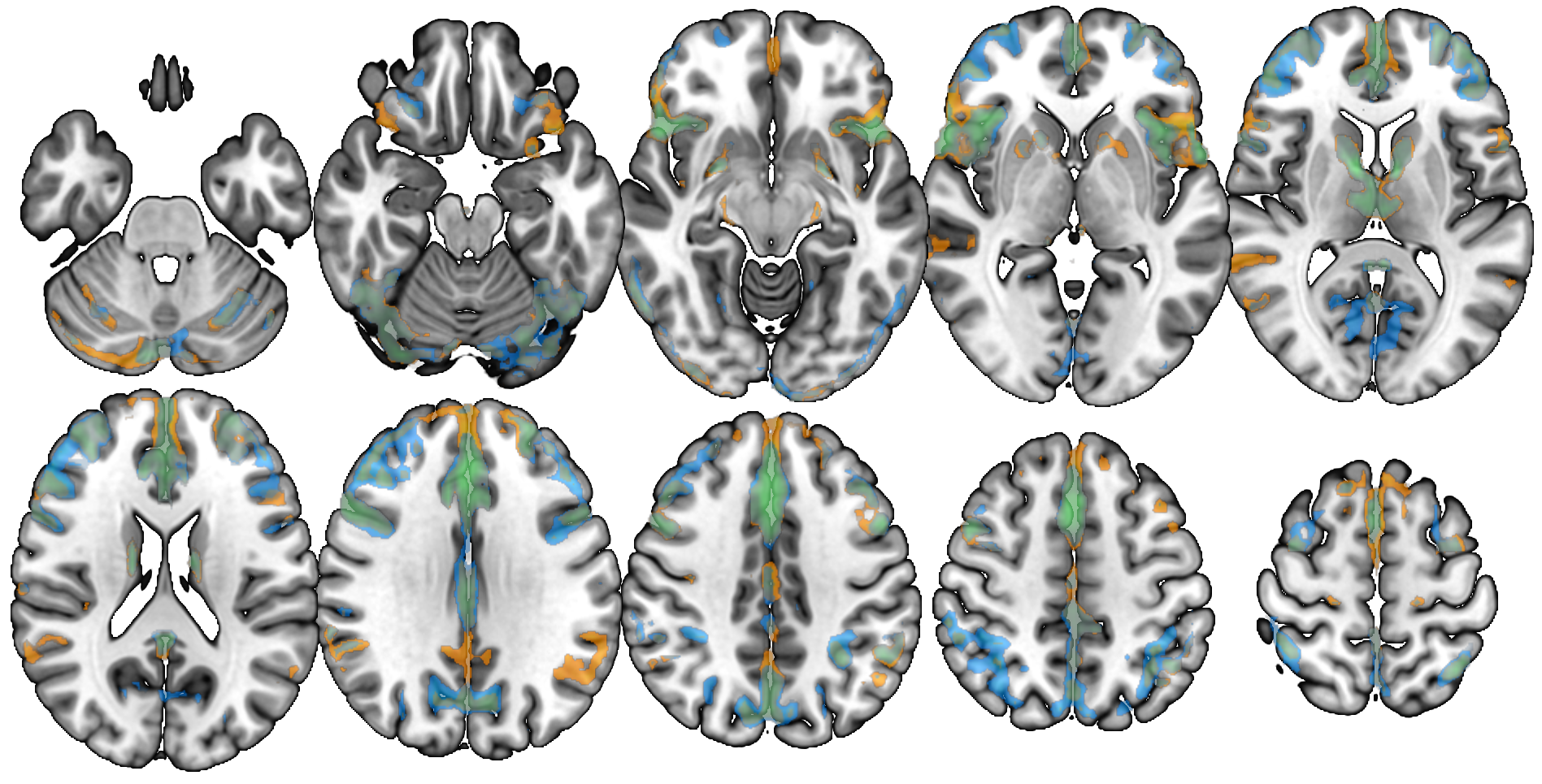


**Supplementary Figure 1. A. Brain regions associated with MDD (cool colors) and emotion-regulation (warm colors) as well as the overlap (green; see also Fig.1) between the two using Neurosynth data. B. Positive connectivity of MDD-related (cool colors) and of emotion regulation-related (warm colors) seed regions (that are presented in the Supplementary Figure 1A), as well as the overlap (green) between the two in the MDDAD data.**

**References**

[1] Komulainen E, Glerean E, Heikkilä R, Nummenmaa L, Raij TT, Isometsä E, et al. Escitalopram enhances synchrony of brain responses during emotional narratives in patients with major depressive disorder. Neuroimage 2021;237:118110.

[2] Komulainen E, Glerean E, Meskanen K, Heikkilä R, Nummenmaa L, Raij TT, et al. Single dose of mirtazapine modulates whole-brain functional connectivity during emotional narrative processing. Psychiatry Res Neuroimaging 2017;263:61-9.

[3] Weigand A, Horn A, Caballero R, Cooke D, Stern AP, Taylor SF, et al. Prospective Validation That Subgenual Connectivity Predicts Antidepressant Efficacy of Transcranial Magnetic Stimulation Sites. Biol Psychiatry 2018;84(1):28-37.

[4] Montgomery SA, Åsberg M. A new depression scale designed to be sensitive to change. Br J Psychiatry 1979;134:382-9.

[5] First MB, Spitzer RL, Gibbon M, Williams JBW. (SCID-I/P) Structured Clinical Interview for DSM-IV-TR Axis I Disorders, Research Version, Patient Edition*.* New York: Biometrics Research, New York State Psychiatric Institute; 2002
